# Double-Tap Interaction as an Actuation Mechanism for On-Demand Cueing in Parkinson’s Disease

**DOI:** 10.3390/s19235167

**Published:** 2019-11-26

**Authors:** Dean Sweeney, Leo R. Quinlan, Margaret Richardson, Pauline Meskell, Gearóid ÓLaighin

**Affiliations:** 1 Electrical & Electronic Engineering, School of Engineering, NUI Galway, University Road, H91 HX31 Galway, Ireland; dean.sweeney@nuigalway.ie (D.S.); gearoid.olaighin@nuigalway.ie (G.Ó.); 2 Human Movement Laboratory, CÚRAM Centre for Research in Medical Devices, NUI Galway, University Road, H91 HX31 Galway, Ireland; 3 Physiology, School of Medicine, NUI Galway, University Road, H91 W5P7 Galway, Ireland; 4 Neurology Department University Hospital Limerick, Dooradoyle, V94 F858 Limerick, Ireland; margaret.richardson@hse.ie; 5 Department of Nursing and Midwifery, University of Limerick, Castletroy, V94 X5K6 Limerick, Ireland; Pauline.Meskell@ul.ie

**Keywords:** cueing, freezing of gait, Parkinson’s disease, on-demand, self-activation, double-tap gesture, electrical stimulation

## Abstract

Freezing of Gait (FoG) is one of the most debilitating symptoms of Parkinson’s disease and is an important contributor to falls. When the management of freezing episodes cannot be achieved through medication or surgery, non-pharmacological methods, such as cueing, have emerged as effective techniques, which ameliorates FoG. The use of On-Demand cueing systems (systems that only provide cueing stimuli during a FoG episode) has received attention in recent years. For such systems, the most common method of triggering the onset of cueing stimuli, utilize autonomous real-time FoG detection algorithms. In this article, we assessed the potential of a simple double-tap gesture interaction to trigger the onset of cueing stimuli. The intended purpose of our study was to validate the use of double-tap gesture interaction to facilitate Self-activated On-Demand cueing. We present analyses that assess if PwP can perform a double-tap gesture, if the gesture can be detected using an accelerometer’s embedded gestural interaction recognition function and if the action of performing the gesture aggravates FoG episodes. Our results demonstrate that a double-tap gesture may provide an effective actuation method for triggering On-Demand cueing. This opens up the potential future development of self-activated cueing devices as a method of On-Demand cueing for PwP and others.

## 1. Introduction

Parkinson’s disease (PD) is the second most common neurodegenerative disease in the developed world. An estimated 1.2 million people are currently living with PD in the EU [1]. Movement disturbances that worsen with the progression of PD are typical characteristics of the disease. Although mainly present in more advanced stages of the disease, Freezing of Gait (FoG) is one of the most debilitating motor-related disturbances [2]. FoG is often described by people with Parkinson’s (PwP) as having their feet “glued to the ground”, despite their intention to walk [3]. The presence of FoG is an important contributor to falls in PwP [4,5,6] and is a significant contributing factor to hospitalization and nursing home admissions [7,8,9].

Within the PD community, cueing is emerging as an effective technique to ameliorate FoG [10,11]. Cueing is defined as the use of external sensory stimuli, providing temporal and/or spatial information to facilitate gait initiation and continuation [12]. The literature extensively reports three different types of cueing modalities: visual, auditory, and somatosensory cueing, each type reflecting the specific stimulus modality (i.e., visual cueing provides visual stimuli).

The majority of cueing systems presented in the literature provide cueing continuously throughout the gait cycle, regardless of whether FoG is present or not. However, recent technological solutions enable cueing systems to provide cueing stimuli only when needed (e.g., during a FoG episode) [13]. These types of systems are termed ‘On-Demand’ cueing systems [14]. During extended periods of usage, On-Demand cueing systems may offer advantages over continuous cueing systems: increased adherence (cueing may be perceived as less annoying/distracting) and increased efficacy (due to reduced habituation) [13]. However, the development of On-Demand cueing systems offers one key technical challenge—an effective actuation method for triggering the onset of cueing is required.

Current solutions include the use of autonomous real-time FoG detection algorithms to trigger cueing when FoG is detected. Cueing systems which utilise these algorithms are termed ‘Intelligent’ cueing systems and are the most common method for triggering On-Demand cueing [15]. The effectiveness of these systems relies on the ability of the FoG detection algorithm to accurately detect a FoG episode with minimum latency (time between the occurrence of a FoG episode and the triggering of cueing) [16]. A comparison of validity measures in terms of sensitivity, specificity and latency suggests that Intelligent cueing systems can accurately detect FoG episodes [17,18,19,20,21,22,23]. However, one should be cautious when interpreting the reported performance of these studies as, in many cases, they may lack a proper ecological validation. Some FoG detection algorithms may also require individual calibration to achieve high validity performance, which has practical significance for applications in clinical practice [17].

Although its aptness still remains to be shown in the literature, an alternative to Intelligent cueing is ‘Self-activated’ cueing. During Self-activated cueing, the user self-detects that they are experiencing a FoG episode and the onset of cueing is triggered directly by the user. Unlike an Intelligent cueing system, the development of a Self-activated system is less complex and potentially requires significantly less computational and power resources to trigger the onset of On-Demand cueing. However, the effectiveness of Self-activated cueing systems in terms of sensitivity, specificity, and latency is dependent solely on the user’s ability to self-detect FoG and perform a physical interaction with the system (e.g., press a switch). In 2013, Bunting-Perry et al. investigated the effect of Self-activated visual cueing on FoG episodes [24]. In the study, participants self-activated cueing by pressing an on/off button on a walking stabilizer during FoG episodes. Although the sensitivity, specificity, and latency were not reported and discouraging results were presented, the study did identify a potential barrier to the use of Self-activated cueing. Bunting-Perry suggested that Self-activated cueing can represent a dual-task (walking while additionally performing a secondary task) that may reduce the positive effect of On-Demand cueing on FoG.

The performance of simultaneously executing a primary and a secondary task (i.e., dual tasking) is a frequent and debilitating problem for people with Parkinson’s (PwP) [25]. Both Spildooren et al. and Petterson et al. examined the effect of cognitive dual tasking (walking while performing a secondary cognitive task) during PD gait [26,27]. Results demonstrated a significant effect of cognitive dual tasking on gait and concluded that cognitive dual tasking increases the risk of experiencing FoG episodes. Alternatively, Chen et al. examined the impact of motor dual tasking (walking while performing a secondary motor task) during PD gait [28]. Results showed that motor dual tasking also increased the likelihood of FoG episodes occurring during gait. Moreover, Galletly et al. investigated the effects of both cognitive and motor dual tasking and reported that while a cognitive dual-task caused deterioration in gait parameters, a motor dual-task did not [29].

These findings indicate the potentially harmful effect of cognitive dual tasking on gait in PD. However, the impact of motor dual tasking has presented conflicting results. Interestingly, it has been reported that motor tasks also occupy part of the cognitive resources (i.e., the mental processes involved in the planning, preparation, and execution of a task) [30]. This, in part, may explain the conflicting results. Motor tasks requiring high cognitive resources [28] may have a more significant negative effect on gait than motor tasks requiring lower cognitive resources [29]. Therefore, in situations where a physical interaction with a cueing system may represent dual tasking, it is important that the required interaction can be carried out with minimal attention demand so that the PwP can remain focused on their primary task.

Physical peripheral interactions (defined as a physical interaction with technology that takes place outside the focus of attention [31,32]) may provide a potential solution which minimizes the cognitive load required to interact with a Self-activated cueing system. In order to perform the interaction in the periphery of attention, these interactions avoid physical actions requiring fine motor control or continuous visual attention [33]. As an example, a number of interactive systems have used accelerometers to facilitate peripheral interactions through the recognition of simple hand gestures (movement of the hand to convey information) [32,33].

The purpose of our work was to assess the potential of a double-tap gesture interaction to facilitate Self-activated cueing for PwP. To this end, we assessed (i) if PwP can effectively perform a double-tap gesture in response to the occurrence of a FoG episode, (ii) if a double-tap gesture performed during FoG episodes can be reliably detected using an accelerometer’s embedded gestural interaction recognition function, and (iii) if a double-tap gesture performed during a FoG episode aggravates the FoG episode itself. Therefore, we hypothesized that a simple hand gesture performed by PwP may provide an effective actuation method for triggering the onset of On-Demand cueing with minimal attention demand. The findings of this study may inform biomedical engineers and clinicians who are considering the use of self-actuation to trigger the onset of On-Demand cueing PD.

## 2. Methods

This study was conducted in three phases. In Phase 1, a double-tap detection function was purposed to enable the detection of double-tap gestures performed by PwP. This function was enabled through the analysis of double-tap gesture signals recorded from 19 PwP. The double-tap gesture was defined as double-tapping action of the whole hand, requiring only gross motor control and enabling eyes-free interaction. In Phase 2, the specificity of the proposed double-tap detection function was assessed on eight healthy participants during scripted and unscripted free-living activities. In Phase 3, the validity of the double-tap detection function as a mechanism to activate On-Demand cueing was evaluated with 10 PwP, which had not taken part in Phase 1 of the study.

### 2.1. Participants 

Twenty-nine (22 men and 7 women; mean age 71.5 ± 8.6 years; and mean disease duration 10.9 ± 8.0 years) participants with idiopathic PD enrolled in the study. A further eight (4 men and 4 women; age 65 ± 9.5 years) healthy participants enrolled in Phase 2 of the study. 

Participants were recruited through Galway Parkinson’s Association of Ireland, Ireland, Clare Parkinson’s Support Group, Ireland, National University of Ireland, Galway, Ireland and University Hospital Limerick, Ireland. All the participants were informed about the nature of the study and provided written informed consent for inclusion before they participated in the study. The study was conducted in accordance with the Declaration of Helsinki, and the protocol was approved by the Galway Hospital Research Ethics Committee (ref: CA 890) for Phases 1 and 2 of the study and the Limerick Hospital Research Ethics Committee (ref: 053/18) for Phase 3 of the study.

PD was diagnosed according to the UK Parkinson’s Disease Society Brain Bank criteria. All PwP participants performed the procedures during the medicated “ON-State”. Inclusion criteria for Phase 1 of the study included able to walk either unaided, with one walking stick or elbow crutch. Inclusion criteria of Phase 3 of the study included a Hoehn and Yahr stage score between two and four, able to walk either unaided, with one walking stick or elbow crutch, a MMSE score greater than 24 and a history of FoG in the medicated “ON-State”.

### 2.2. Hardware 

We used a custom-built wearable cueing device for all three phases of the study. The cueing device was a waist-worn voltage-controlled, two-channel, Bluetooth enabled electrical stimulator developed at the NUI Galway. The device was designed to deliver either continuous or On-Demand sensory electrical stimulation (sES) to ameliorate FoG [34].

In Phases 1 and 2 of this study, the cueing capabilities of the cueing device were disabled, and the device was configured to operate only as an inertial measurement data logger. Data logging was facilitated through the cueing device’s on-board tri-axial accelerometer and a microSD card. The tri-axial accelerometer (LIS2DH, STMicroelectronics) was used to measure z-axis (anterior-posterior axis) acceleration magnitudes up to ±8 g with a resolution of 63 mg (1 g = 9.81 m/s^2^) and at a sampling frequency of 400 Hz. The removable microSD card allows access to the stored raw accelerometer data. Thus, the cueing device provided a platform to record the double-tap gesture signals of PwP.

In Phase 3 of this study, the cueing capabilities of the cueing device were enabled. In this mode, the device can be either configured to trigger On-Demand cueing locally by the user (through an embedded double-tap detection function within the LIS2DH tri-axial sensor) or remotely by a clinician (through a custom-built smartphone application, reported in a previous study [35]).

The LIS2DH double-tap detection function provides four registers, which can be configured to enable the detection of specific double-tap signatures. Table 1 highlights the double-tap configurable registers and their range of values. The resolution of these values directly related to the selected sampling frequency and acceleration range of the accelerometer, 400 Hz and ±8 g. The resolution of the Threshold Register is given as the absolute value of the acceleration range (8 g) divided by the maximum register value (127), 63 mg. The resolution of the remaining three double-tap registers is given as one divided by the sampling frequency (400 Hz), 2.5 ms.

**Figure 1:**
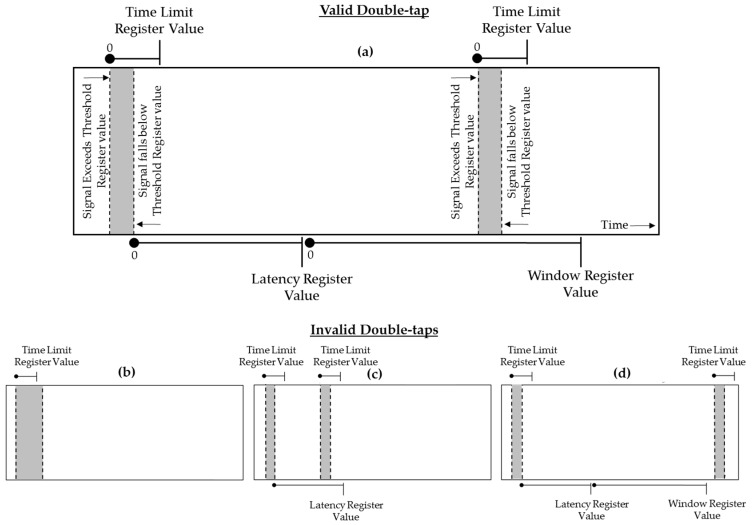
Double-tap signature recognition conditions: (**a**) Valid double-tap due to the first and second-tap signal not exceeding the Time Limit Register value while the second-tap signal exceeds the Latency Register value and occurs before the Window Register value is exceeded; (**b**) Invalid double-tap due to signal not falling below the Threshold Register value before the Time Limit Register value was exceeded; (**c**) Invalid double-tap due to second tap occurring before the Latency Register value was exceeded; (**d**) Invalid double-tap due to second tap occurring after the Window Register value was exceeded.

A valid double-tap signature is detected if its signal conforms to the value in each double-tap register (Figure 1a). The Threshold Register configures the double-tap acceleration (±g) value that must be exceeded for a double-tap to be detected. The Time Limit Register sets the maximum duration of the first and second tap. If any tap exceeds this value, it will not be recognized as a double-tap (Figure 1b). The Latency Register is used to configure the minimum time over which the second tap must occur after the first tap. If the second tap occurs in less time than the Latency Register value, it will not be recognized as a double-tap (Figure 1c). The Window Register configures the maximum time after the Latency Register value in which the second tap must occur. If the second tap occurs after the Window Register value, it will not be recognized as a double-tap (Figure 1d). To permit the configuration of these registers, an accompanying smartphone application facilitated the wireless programming of each register.

**Figure 2:**
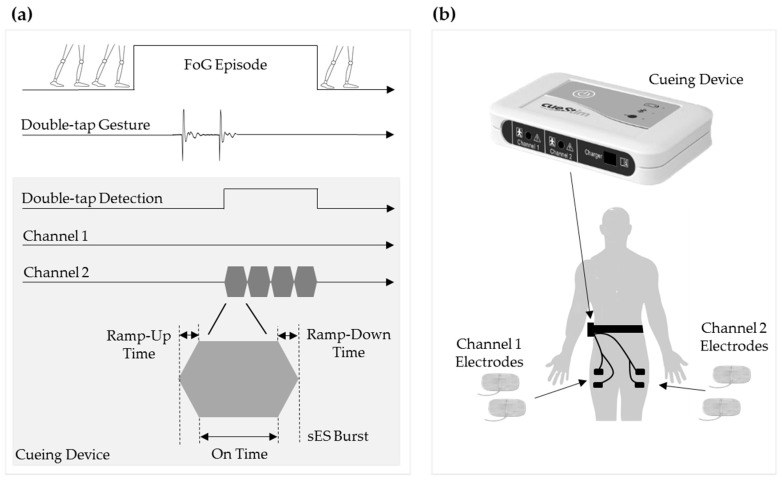
On-demand cueing delivery technique: (**a**) Illustrates the execution of a double-tap gesture in response to the self-detection of a FoG episode and the subsequent successful recognition of the double-tap gesture by the cueing device which immediately triggers the delivery of sES on Channel 2; (**b**) Illustration of a participant wearing the cueing device located in a waist-worn belt holder with Channel 1 and Channel 2 electrodes connected to the right and left anatomical sites of quadriceps muscle.

The successful recognition of a double-tap signature by the LIS2DH double-tap detection function immediately triggers the delivery of four biphasic sES bursts to either Channel 1 and/or Channel 2 of the cueing device (Figure 2a). The removable microSD card provides a platform to record all double-tap detection and cueing events on the device.

Each sES burst consisted of 100 ms Ramp-up time, 500 ms ON time, 100 ms Ramp-down time and 0 ms OFF time. Cueing was facilitated through the use two pairs of 5 × 10 cm skin surface electrodes (Axelgaard PALS Electrodes, Denmark). One pair of electrodes was connected to Channel 1 of the cueing device and placed on the skin surface over two anatomical sites on the right quadriceps muscle (Figure 2b). The other pair of electrodes was connected to Channel 2 of the cueing device and placed on the skin surface over two anatomical sites on the left quadriceps muscle.

The accompanying smartphone application enables a clinician to select which stimulation channel is to be used (i.e., Channel 1 and/or Channel 2) and to adjust the intensity of the sES bursts for each channel. The intensity of the sES burst was set for each participant such that a maximum sensory response was elicited but that the amplitude was not of sufficient intensity to produce a motor or pain response.

A Canon (Tokyo, Japan) Legria HF R46 camera was used to record video during all participant testing in Phase 3 of the study. The video quality was set to capture full HD video at a frame rate of 25 progressive frames per second (17 Mbps).

### 2.3. Phase 1: Double-Tap Detection Function for PwP

Nineteen participants with idiopathic PD completed Phase 1 of the study (13 men and 6 women; 71.9 ± 9.0 years; mean disease duration 9.6 ± 9.2 years). A database of double-tap gesture signals was created using the following procedure. While standing, each participant was required to perform a set of 10 double-tap gestures on the surface of the cueing device. An investigator instructed the participant to perform a double-tap gesture when requested and then return their hand to their side. Each participant received a total of ten requests from the investigator. An initial demonstration was carried out by an investigator to clarify whether the participant understood the procedure. Following the demonstration, the investigator secured the cueing device to the participant’s waist (left or right hip corresponding to the participant’s side of dominant hand) using a waist-worn belt holder. Before beginning the procedure, the participants were allowed to perform up to three double-tap gesture practice attempts. Upon completion of the procedure, a personal computer was used to upload the logged accelerometer signals from the cueing device via a removable micro SD-card.

**Figure 3:**
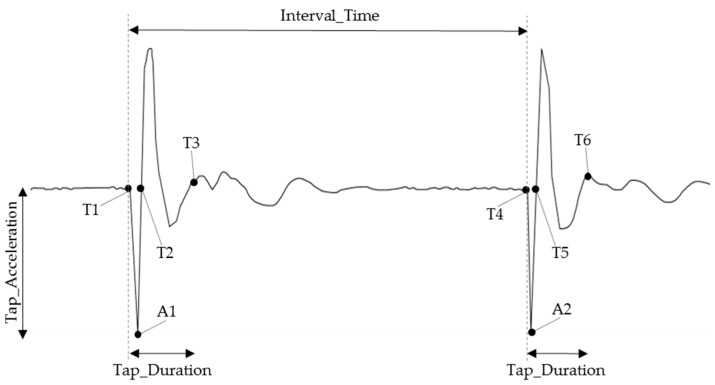
Double-tap gesture signal and key characteristics measurement. T1→T4: Interval_Time; T1→T2 and T4→T5: Tap_Width; T1→T3 and T4→T6: Tap_Duration; A1 and A2: Tap_Acceleration.

Analysis of the double-tap gesture signals was carried out within Matlab (MathWorks, Natick, MA, USA). As illustrated in Figure 3, four double-tap characteristics were measured from each recorded double-tap gesture signal:

- Tap_Acceleration: defined as the maximum acceleration recorded for each tap, measured at A1 and A2.
- Interval_Time: defined as the time between the beginning of the first tap (T1) and the beginning of the second tap (T4).
- Tap_Width: defined as the base width of the initial impact spike of the first and second tap, measured between T1→T2 and T4→T5.
- Tap_Duration: specifies the total duration of a tap as measured from the initial point of impact until a point in which the signal regress to its post tap state, measured between T1→T3 and T4→T6.

To tailor the LIS2DH double-tap detection function to the needs of PwP, we statistically analyzed these four double-tap characteristics to infer a range of values that are representative for all PwP. The LIS2DH double-tap registers where then configured using Equations (1)–(4):

Threshold Register ≤ Tap_Acceleration_min_ (1)

Time Limit Register ≥ Tap_Width_max_ (2)

Interval_Time_min_ ≥ Latency Register ≥ (Tap_Duration_max_ − Tap_Width_max_) (3)

Window register ≥ (Interval_Time_max_ − Tap_Duration_max_) (4)

where Tap_Acceleration_min_ is inferred from the mean value of all the participants’ recorded minimum Tap_Acceleration value; Tap_Width_max_ is inferred from the mean of all participant’s recorded maximum Tap_Width value; Tap_Duration_max_ is inferred from the mean of all participant’s recorded maximum Tap_Duration value; Interval_Time_max_ is inferred from the mean of all participant’s recorded maximum Interval_Time value. Interval_Time_max_ and Interval_Time_min_ are inferred from the mean of all participant’s recorded maximum and minimum Interval_Time value.

### 2.4. Phase 2: Specificity Testing with Health Controls

Eight healthy volunteers (4 men and 4 women; 54.38 ± 13.90 years) participated in Phase 2 of the study. Using the double-tap register values derived from Phase 1 of the study, the specificity of the double-tap detection function was assessed. To ensure that the specificity of the double-tap detection function was rigorously tested, participants were required to perform both scripted and unscripted free-living activities in their homes and in their community while wearing the cueing device. Throughout the procedure, the cueing device was secured to the participant’s waist (left or right hip corresponding to the participant’s side of dominant hand) using a waist-worn belt holder. To reduce possible biasing, the participants were only instructed that the cueing device was used to record their movement.

The specificity of the double-tap detection function was initially tested in a limited number of situations, which were representative of free-living activities and that may result in the accidental recognition of double-taps by the device. The scripted activities are presented in Table 2. These activities were selected as they could potentially result in unwanted forces being applied to the z-axis (anterior–posterior axis) of the cueing device. To ensure that all of the scripted activities were completed, participants were supervised by a researcher. Following the scripted activities, participants continued to wear the cueing device under unsupervised conditions for two days (>8 h per day). During this period, participants carried out their unscripted free-living activities, such as driving and shopping. A diary was provided to each participant and they were required to record their activities on an hourly basis. Upon completion of the procedure, a personal computer was used to upload both the logged accelerometer signals and the output of the double-tap detection function from the cueing device. The output from the double-tap detection function was either high if the function recognized a double-tab gesture, or low if it did not.

In order to validate the specificity Equation (5), its calculation parameters were assessed: False positive (FP), a double-tap is not performed, but one is recognized by the cueing device; True negative (TN), a double-tap is not performed, and one is not recognized by the cueing device.

Specificity = TN/(TN + FP)(5)

### 2.5. Phase 3: Validity Testing with PwP

Ten participants (9 men and 1 woman; 70.6 ± 7.7 years; mean disease duration 13.4 ± 4.0 years; mean FOGQ score 15 ± 1.55) with idiopathic PD completed Phase 3 of the study (Table 3). All assessments were carried out on participants while in the medicated “On-State” (self-reported by the participant). All the participants identified a one-minute-walking-task within their home which usually elicited FoG episodes. The common features in each of the walking tasks performed were (i) performing a turn during walking (ii) walking through a doorway (iii) walking across a room and walking in a corridor/hallway. During testing, participants performed the walking task for both Self-activated and Clinician-activated On-Demand cueing. Prior to the walking task with Self-activated cueing, participants were instructed to perform a double-tap gesture only when they experienced a FoG episode. During walking tasks with Clinician-activated cueing, participants were instructed not to perform a double-tap gesture during the walking task. Instead, when the clinician (Parkinson’s Disease *Nurse* Specialist) identified a FoG episode, the clinician would remotely activate On-Demand cueing using the smartphone application.

An initial demonstration was carried out by an investigator to assist the participant in understanding the procedure. Following the demonstration, the investigator secured the cueing device to the participant’s waist (left or right hip, corresponding to the participant’s side of dominant hand) using a waist-worn belt holder.

Each participant performed the walking task 12 times. Six times during Self-activated cueing (three times while cueing is delivered to the left quadriceps and three times while cueing is delivered to the right quadriceps) and six times during Clinician-activated cueing (three times while cueing is delivered to the left quadriceps and three times while cueing is delivered to the right quadriceps). The order of each walking task was randomly assigned using block randomization [35]. As part of the testing, the participants’ lower limbs were video-recorded with the HD camera as they performed each walking task.

**Figure 4:**
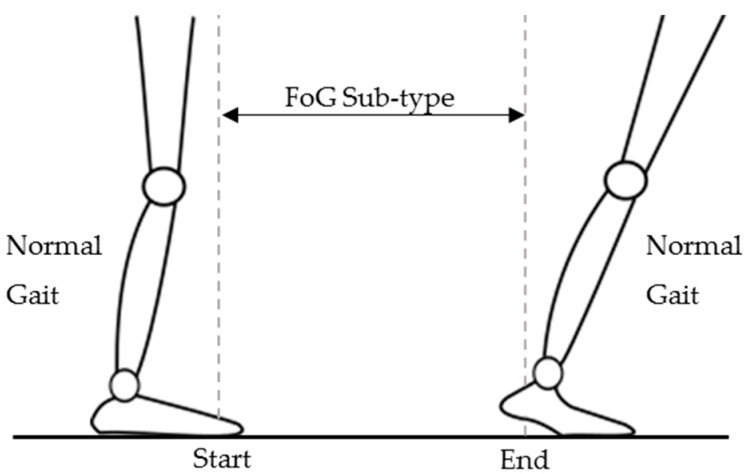
Start and end markers of a FoG episode.

During a post-experiment video analysis, an experienced clinician (Parkinson’s Disease *Nurse* Specialist) labeled the start and end times of each FoG episodes identified within each participant’s video recording. In addition, the clinician labeled each sub-type of FoG, if present: (i) purely akinesia form (no motion of the person’s legs is observed) (ii) ‘‘tremble in place’’ form (inability of the person to step with their legs trembling at a frequency of 2 to 4 Hz) and (iii) ‘‘shuffling’’ form (spontaneous increase in cadence and decrease in step length). When a FoG episode was identified, the start of that FoG episode was taken as the last point in time at which the participant was in the foot-flat stage of the gait cycle, pre-FoG episode, Figure 4. The end of that FoG episode was taken as the point in time at which the participant was in the heel-off stage of the gait cycle for the first successful step, post-FoG episode, Figure 4.

The clinician who performed the labeling was also physically present during the recording sessions. However, prior to labeling (i) all the videos were edited to remove all audio and (ii) the order of the 12 walking tasks was randomly shuffled. In this sense, it is important to note that the clinician was unaware of the type (Self-activated or Clinician-activated) of On-Demand cueing being applied in each video.

A researcher labeled the end time of each double-tap gesture identified within the participant’s video. To assist in the process, the recorded movement signals logged on the microSD card were analyzed within Matlab to identify each double-tap gesture performed. Video and inertial signals were synchronized based on a predefined event (5 single taps performed at 1 Hz) which can be identified in both the video recordings and the movement signals.

The combination and analyses of both the clinician and researcher annotations enabled the evaluation of the viability of a double-tap gesture as an actuation mechanism to trigger On-Demand cueing in terms of

- the latency and sensitivity of PwP to perform a double-tap gesture in response to the occurrence of FoG;
- the sensitivity and specificity of the proposed double-tap detection function;
- the impact of performing a double-tap gesture on FoG episode durations during On-Demand cueing;

#### 2.5.1. Latency and Sensitivity of Performing a Double-tap

To validate whether PwP can adequately perform a double-tap gesture in response to the occurrence of a FoG episode, both latency and sensitivity were assessed. Latency was calculated as the time between the start of a FoG episode (identified using the clinician video annotation) and the completion of a responding double-tap gesture (identified by the researcher). The sensitivity Equation (6) was calculated using the following parameters:

- True positive (TP), a FoG episode occurs, and a double-tap is performed;
- False negative (FN), a FoG episode occurs, and a double-tap is not performed.

Sensitivity = TP/(TP + FN)(6)

In some cases, a double-tap gesture may not have been performed in response to the occurrence of some FoG episodes, because the participant exited the FoG episode before they could perform a double-tap gesture. Such events could be classified as FN, however it is probably that the duration of the FoG episode was shorter than the time it takes the participant to perform a double-tap. Therefore, to reduce the number of FN misclassifications, FoG episodes with a duration less than the participant’s mean latency time were excluded from the sensitivity analysis.

#### 2.5.2. Sensitivity and Specificity of Double-tap Detection Function

To validate if the double-tap detection function can accurately detect a double-tap gesture during a FoG episode, its sensitivity and specificity were calculated using Equations (5) and (6), respectively.

From the analysis of the researcher’s annotations and the logged events on the cueing devices’ microSD card, the following parameters were assessed:

- True positive (TP), a double-tap is performed, and it is recognized by the device;
- False positive (FP), a double-tap is not performed, and it is recognized by the device;
- True negative (TN), a double-tap is not performed, and it is not recognized by the device;
- False negative (FN), a double-tap is performed, and it is not recognized by the device.

#### 2.5.3. Impact of Double-Tap Gestures on FoG Episode 

To asses if the action of performing a double-tap gesture aggravates FoG episodes, the duration and number of FoG episodes occurring with Self-activated cueing was compared to the duration and number of FoG episodes occurring with Clinician-activated cueing. FoG episodes with a duration less than the participant’s mean latency value were not excluded from the analysis. However, only TP parameters were analyzed (i.e., a FoG episode occurs, and a double-tap is performed and cueing is activated or the clinician remotely activates cueing using the smartphone application).

#### 2.5.4. User Experience

Upon completion of the walking tasks, a participant questionnaire was completed, which incorporated a standard 100 mm Visual Analogue Scale (VAS) and a Face Pain Rating Scale to assess the perceived comfort/pain level associated with the sES cueing. This questionnaire included questions regarding the suitability of the intensity of sES cueing, the suitability of the location of the sES cueing device and the perceived difficulty level of performing a double-tap gesture on the sES cueing device.

### 2.6. Statistical Analysis

A preliminary *t*-test was run to test for possible differences between the left and right limbs. As no significant difference between the duration of FoG episodes was revealed by the analyses, we used the mean of the two limbs for each participant in the subsequent analyses. An independent samples *t*-test was conducted to assess whether there were differences between the mean duration of FoG episodes of participants during Self-activated and Clinician-activated On-Demand cueing. Statistical calculations were performed with SPSS Version 24 (IBM Corporation, New York, USA). A *p*-value < 0.05 was considered statistically significant in all cases.

## 3. Results

### 3.1. Phase 1: Double-Tap Detection Function for PwP

**Figure 5:**
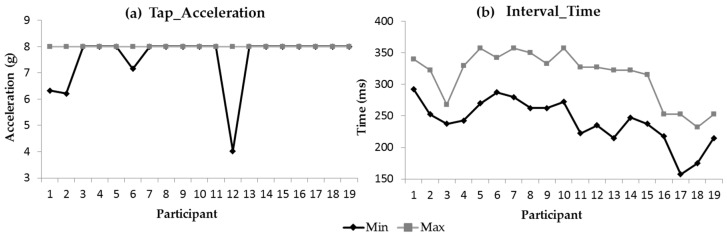

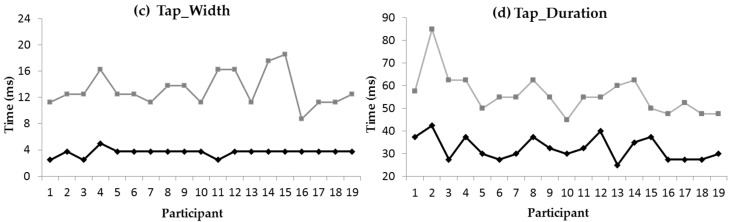
Participants’ individual double-tap characteristics (minimum and maximum recorded values).

A total of 190 double-tap signals were recorded. The data show that all participants were capable of performing a double-tap gesture. Analyses of these signals within Matlab identified the minimum and maximum values for the four double-tap characteristics (Figure 5).

Based on the results in Figure 5, the mean of all the participants’ recorded minimum Tap_Acceleration value was 7.56 g ± 1.03 g. From the 380 single-tap signals (190 double-tap) only seven taps failed to produce an acceleration equal to or greater than 8 g. The mean of all participans’ recorded maximum Tap_Width value and Tap_Duration value was 13.49 ms ± 3.24 ms and 56.18 ms ± 8.99 ms, respectively. The mean of all participants’ recorded maximum and minimum Interval_Time value was 313.82 ms ± 40.68 ms and 241.18 ms ± 35.67 ms, respectively.

To generalize the extracted double-tap characteristics to values that are representative of the double-tap feature values of the majority of PwP, the 99.7 rule, also known as the empirical rule (three standard deviations), was implemented. For example, it can be inferred that 99.7% of our sample population will have a minimum Tap_Acceleration value equal to

Tap_Acceleration_min_ = mean − (3 × SD) = 7.56 g − (3 × 1.03 g) = 4.47 g.

Using this rule, we can infer that the PwP population will have the double-tap characteristics summarized in Table 4.

The LIS2DH double-tap registers were then configured based on Equations (1)–(4) and register resolution values (Table 1):

Threshold Register = Tap_Acceleration_min_ = 4.44 g.

Time Limit Register = Tap_Width_max_ = 20 ms.

Latency Register = (Tap_Duration_max_–Tap_Width_max_) = 62.5 ms.

Window Register = (Interval_Time_max_–Tap_Duration_max_) = 362.5 ms.

### 3.2. Phase 2: Specificity Testing with Health Controls

The analysis of the logged output of the double-tap detection function revealed that on no occasion, a double-tap signal was detected during the scripted activities (i.e., FP = 0). During the scripted activities, the highest acceleration recorded was during activity number 16 (while sitting, removing an item from your front pocket) at 3.58 g.

A total of 132 h of unscripted activities were recorded. The analysis of the logged output of the double-tap detection function revealed that only on two occasions during the 132 h of unscripted activities was a double-tap gesture detected. Therefore, using Equation (5), the double-tap detection function achieved a specificity of 100% during scripted activities and ~100% during unscripted activities with healthy controls.

### 3.3. Phase 3: Validity Testing with PwP

From the 10 participants that completed Phase 3 of the study, four participants predominantly displayed a ‘‘shuffling’’ form of FoG, three predominantly displayed a ‘‘akinetic’’ form of FoG and one predominantly displayed a ‘‘tremble in place” form of FoG. Two participants displayed both ‘‘akinetic’’ and ‘‘tremble in place” forms of FoG.

An independent samples *t*-test showed that there was no significant difference between the ages of participants in Phase 1 and Phase 3 (71.9 ± 9.0 years versus 70.6 ± 7.7 years, *p* = 0.708). The statistical analysis of the difference between the disease duration of participants in Phase 1 and Phase 3 also showed no significant difference (9.6 ± 9.2 years versus 13.4 ± 4.0 years, *p* = 0.232).

#### 3.3.1. Latency and Sensitivity of Performing a Double-Tap

Table 5 summarizes participant latency times associated with performing a double-tap gesture as an actuation mechanism to trigger cueing. The mean latency, reported as the time between the occurrence of a FoG episode and its detection by the participant through the performance of a double-tap gesture on the cueing device, is given as 1.42 s ± 1.17 s.

The lowest mean latency was recorded by participant seven, 0.79 s ± 0.69 s and the highest mean latency was recorded by participant ten, 2.34 s ± 1.94 s.

A total of 176 FoG episodes were identified through video analysis. Participants correctly identified 138 episodes and failed to identify 38, resulting in a mean sensitivity of 76.87% ± 13.58%. Participant three achieved the highest sensitivity (95.45%), while participant two achieved the lowest sensitivity, 46.15%.

In addition, participants mis-identified 18 FoG episodes through double-tap gestures (FP = 18) when no FoG episode occurred. Participant one performed six FPs, participant six performed five FPs and the remaining FPs were attributed to participants three, four, seven, eight and nine. A total of 67 FoG episodes were removed from analysis because their durations were shorter than the participants’ latency times (Table 6). The mean duration of all the removed FoG episodes was 0.76 s ± 0.58 s.

#### 3.3.2. Sensitivity and Specificity of Double-tap Detection Function

Table 7 reports the sensitivity of the cueing device’s double-tap detection function for individual participants. Taking into account the parameters for calculating sensitivity in Equation (6), the number of TP and FN are also presented. The mean sensitivity in recognizing a double-tap gesture performed by participants was 96.39% ± 4.13%. In the absence of all participants performing a double-tap gesture, on no occasion did the cueing device incorrectly detect a double-tap (FP = 0). Therefore, using Equation (5), the double-tap detection function obtained a specificity of 100% for all participants.

The nine FN (non-detected double-tap gestures) resulted from seven taps that failed to exceed the double-tap Threshold register value (4.47 g) and two taps that exceeded the double-tap Window register value (363 ms).

#### 3.3.3. Impact of Double-tap Gestures on FoG Episode

A total of 136 FoG episodes that occurred during Self-activated cueing and a further 140 FoG episodes that occurred during Clinician-activated cueing were analyzed. Table 8 highlights the time duration of FoG episodes using Self-activated cueing versus Clinician-activated cueing.

An independent samples *t*-test showed that there was a significant difference between the mean duration of FoG during Self-activated cueing and the mean duration of FoG during Clinician-activated cueing (4.20 s ± 2.49 s versus 3.35 s ± 2.18, *p* = 0.005). However, an individual analysis revealed that for eight of the participants, the duration of FoG episodes was not significantly different between Self-activated and Clinician-activated cueing.

An independent samples *t*-test showed that the mean duration of FoG episodes for participant 1 significantly increased from 1.70 s ± 0.23 s (Clinician-activated) to 2.94 s ± 0.67 s (Self-activated), *p* = 0.03. Furthermore, the mean duration of FoG episodes for participant 10 significantly increased from 4.85 s ± 2.02 s (Clinician-activated) to 8.01 s ± 3.18 s (Self-activated), *p* = 0.01.

Interestingly, Self-activated cueing resulted in less FoG episodes occurring for some participants in comparison to Clinician-activated cueing. Participants 5 and 9 had a substantially lower number of FoG episodes (~50% reduction) occurring during Self-activated cueing. An independent samples *t*-test showed that the mean number of FoG episodes occurring during Clinician-activated cueing (24.40 ± 16.23) was not significantly different from the mean number of FoG episodes occurring during Self-activated cueing (20.30 ± 10.41), *p* = 0.51.

#### 3.3.4. User Experience

Participant VAS scores ranged from 3.5 cm (mildly comfortable) to 9.4 cm (very comfortable) with a mean score of 7.93 cm (comfortable) for the perceived comfort of sES cueing. Eight participants give the Face Pain Rating Scale a score of 0 (no pain), with participants 6 and 10 both giving a score of 2 (mild, annoying pain) for the pain level of sES cueing. When asked if the sES cueing intensity level was high enough during the testing, nine of the participants responded with “Yes”. Participant 2 responded “Unsure”. Nine participants responded with “Yes” when asked if the double-tap was easy to perform. Participant 10 responded “Unsure”. When asked if they would prefer the location of the sES cueing device at a different location, all participants responded with “No”.

## 4. Discussion

This paper is the first to investigate the viability of a double-tap gestural interaction as an actuation mechanism for On-Demand cueing in PD. The current results demonstrate that a double-tap gesture may provide an effective actuation method for triggering the delivery of On-Demand cueing.

### 4.1. Phase 1: Double-tap Detection Function for PwP

In Phase 1, we reported four double-tap characteristics (tap acceleration, tap interval time, tap width and tap duration) from a cohort of 19 PwP. Although, the number of participants in Phase 1 was limited, the authors inferred a set of LIS2DH double-tap register values based on Equations (1)–(4). This enabled an initial validation of a double-tap gestural interaction modality as an actuation mechanism for On-Demand cueing in PD. An analysis revealed that the mean acceleration for each participant’s lowest recorded single-tap acceleration value was 7.56 g ± 1.03 g. In comparison, analysis of the Daphet FoG dataset (wearable acceleration sensor data on 10 PwP with FoG) [36] revealed that during PD gait and activities of daily living, the maximum anterior-posterior axis accelerations recorded on a waist worn sensor were 3.36 g ± 0.90 g. These findings suggest that the acceleration associated with a double-tap gesture by PwP on a waist worn sensor is significantly higher than the background accelerations produced during gait or activities of daily living. Furthermore, we demonstrated that an accelerometer with an acceleration range ±8 g is suitable to record the double-tap gestures of PwP.

In addition to the tap acceleration value, the tap interval time is an important double-tap characteristic. The tap interval times extracted in Phase 1 of the study were similar to the tap interval times presented by Yahalom et al. [37]. Yahalom et al. investigated the fastest speed at which PwP (32 men and 19 women; 66.3 ± 9.1 years; mean disease duration 7.5 ± 5.7 years) can perform hand tapping (tapping with fingers by moving their wrist). A mean interval time of 228 ms was reported by Yahalom in contrast to the mean interval time of 274 ms reported in our study. The slower mean interval time of 274 ms reported in our study may reflect the instruction given to the participants. Yahalom et al. instructed the participants to perform taps at their fastest pace. While in our study participants were instructed to perform taps at a comfortably fast pace.

Interestingly, Yahalom et al. classified their participants into four subtypes (tremor predominant, FoG predominant, akinetic-rigid, and unclassified). Interestingly, no significant difference between groups were reported. This suggests that the tap interval time of different cohorts of PwP (i.e., PwP with and without FoG) may not be significantly different. Furthermore, our choice to use an acceleration sampling frequency of 400 Hz (as recommended by the LIS2DH manufacturers for double-tap recognition) has been demonstrated to be sufficient to record the double-tap gestures of PwP.

Using the 99.7 rule, the inferred set of LIS2DH double-tap register values derived in Phase 1 (Threshold Register = 4.44 g, Time Limit Register = 20 ms, Latency Register = 62.5 ms, and Window Register = 362.5 ms) were proposed by the authors as suitable to achieve a high double-tap detection sensitivity rating.

### 4.2. Phase 2: Specificity Testing with Health Controls

In Phase 2, we reported that the double-tap detection function achieved a specificity of 100% during scripted activities and ~100% during unscripted activities with healthy controls. It was our view that healthy controls are typically more active in their daily life in comparison to PwP with FoG. Therefore, by using healthy controls, the specificity could be more rigorously tested with a greater set of movement activities over a shorter period. Only on two occasions during the 132 h of unscripted activities was a double-tap gesture detected incorrectly. The precise activity which triggered a double-tap detection is unclear due to the participants carrying out multiple activities during the period of the detection.

### 4.3. Phase 3: Validity Testing with PwP

In Phase 3, it was firstly shown that participants performed a double-tap gesture in response to the occurrence of FoG with an average latency of 1.42 s ± 1.17 s. Interestingly, the performance of the double-tap gesture, in terms of latency, is comparable to the latency of some Intelligent cueing systems (<2 s [17], 1.1 s [21] and 1 s [22]). Nevertheless, the requirement for a latency value of less than 2 s is up for discussion. From the combined analysis of subjective FoG questionnaires (FOG-Q and GFQ [38]), approximately 40% of FoG episodes last between 1and 2 s [39,40,41]. However, mild FoG episodes that last such a short time may not be troublesome for PwP as they may not interfere with their daily lives [13,42]. Therefore, based on our results, it is our view that PwP can perform a double-tap gesture in a sufficient time to trigger On-Demand cueing to relieve potential troublesome FoG episodes. Furthermore, this was the first time that the study participants performed a double-tap gesture in response to the occurrence of FoG. Therefore, with increased practice, it may be possible to further reduce the latency time between the start of a FoG episode and the completion of a responding double-tap gesture.

Secondly, in Phase 3, it was shown that participants performed a double-tap gesture in response to the occurrence of FoG with an average sensitivity of 76.87% ± 13.58%. However, some participants achieved a sensitivity of 95%, while others only achieved a sensitivity of 46%. These disparate results may reflect the inability of some PwP to self-detect FoG. However, due to the short protocol and limited amount of training, some participants may have simply forgotten to perform a double-tap gesture during some FoG episodes. In addition, the average FoG episode durations that were not identified by participants were short. Indeed, 71% of them had FoG episode durations that were one standard deviation away from the participant’s mean latency time.

Although the sensitivity reported in this paper was lower than the reported sensitivity of some Intelligent cueing systems, 82.2%–97% [17,18,19,20,21,22,23], one should be always cautious when interpreting performance (in terms of sensitivity and specificity) of On-Demand cueing systems. In particular, one should consider the study protocol design (e.g., home versus laboratory environment; scripted versus unscripted protocols), system design (e.g., one device versus a set of combined devices), sample size, the definition of FoG adopted and the type of FoG presented by participants within the study [17]. Our study protocol was based in a home environment with participants defining their own walking task. Such unscripted tasks may provide a more rigorous test of sensitivity and specificity in comparison to scripted tasks in a laboratory environment. In addition, the participants in this study displayed a wide range of FoG sub-types (i.e., ‘‘shuffling’’, ‘‘tremble in place’’ and “akinetic”) which, again, may provide a more rigorous test of sensitivity and specificity in comparison to a cohort of participants all with similar FoG sub-types.

Furthermore, in Phase 3, it was shown that the double-tap detection function derived in Phase 1 of the study can achieve a specificity of 100% and a sensitivity of 96%. Our choice to configure the accelerometer’s (LIS2DH) embedded double-tap detection function based on the characteristics extracted from a database of double-tap signals acquired from PwP, aimed to increase the specificity and sensitivity of the detection function.

Although the specificity of the double-tap detection function was only assessed on PwP during their home-based walking task (Phase 3 of the study), the specificity was also assessed on healthy controls (Phase 2 of the study). The overall results observed suggest that an accelerometer’s embedded gestural interaction function can potentially be used as a viable method to reliably detect the double-tap gesture of PwP during a FoG episode.

The final results from Phase 3 show that performing a double-tap gesture did not significantly aggravate FoG episode durations for most participants. However, for two participants, performing a double-tap gesture significantly increased the participant’s duration of FoG episodes. Indicating that for some PwP a double-tap gesture may represent a dual-task, that requires a significant cognitive load to execute. However, the limited amount of training and the relative unfamiliarity of the participants in performing the required double-tap gesture (before participating in this study) may be attributed to an increased cognitive load. As the PwP becomes more learned to the action of a double-tap gesture, the cognitive load requirements may decrease as the action becomes more natural and instinctive.

Interestingly, Self-activated cueing resulted in less FoG episodes occurring for some participants in comparison to Clinician-activated cueing, although the mean number of FoG episodes occurring was not statistically different between Self-activated cueing and Clinician-activated cueing. These findings further demonstrate that a double-tap gesture performed during a FoG episode does not significantly aggravate FoG episodes.

### 4.4. Limitations

In the current study, a small cohort of 19 PwP was used to infer a double-tap detection function, yet the double-tap detection function achieved a specificity of 100% and a sensitivity of 96%. However, it is important to acknowledge that the sensitivity was evaluated on a small cohort (10 participants). As the symptoms of PD, such as dexterity impairment and motor deficits like bradykinesia (characterized by slowness and decreased amplitude of movement) [43], are experienced by PwP to different degrees, our database of double-tap signals may be of insufficient size to infer the double-tap characteristics that are representative for all PwP. This may in part account for the nine non-detected double-tap gestures, which resulted in a sensitivity value of 96%. In addition, no participants within the study displayed freezing of the upper limb (FOUL). Although FOUL is not correlated to FoG [44], it is possible that during a FoG event, PwP with FOUL may be unable to perform a double-tap gesture.

As would be expected in a new area of research, there remains a large number of directions for future work on this topic. Predominantly, there remains a need for a more thorough validation of Self-activated cueing systems, as work to date has involved low participant numbers.

## 5. Conclusions

PwP can effectively perform a double-tap gesture in response to the occurrence of a FoG episode with an average latency of 1.42 s ± 1.17 s and sensitivity of 76.87% ± 13.58%. A double-tap gesture performed during a FoG episode can be reliably detected using an accelerometer’s embedded gestural interaction recognition function with a specificity and sensitivity of 100% and a 96%, respectively. For some PwP, a double-tap gesture performed during a FoG episode does not significantly aggravate FoG episode duration or frequency. Collectively, these three key findings suggest the possible feasibility of Self-activated cueing as a method of On-Demand cueing, which can be easily implemented and focuses on empowering PwPs to better self-manage their FoG episodes.

## Figures and Tables

**Table 1:** The tri-axial accelerometer (LIS2DH) double-tap register value ranges for a sampling frequency of 400 Hz with a full-scale value of ±8 g.

Register	Register Value	Resolution	Range
Threshold	0–127	63 mg	0–±8 g
Time Limit	0–127	2.5 ms	0–318 ms
Latency	0–255	2.5 ms	0–638 ms
Window	0–255	2.5 ms	0–638 ms

**Table 2:** Scripted activities and number of times performed during specificity testing.

No.	Activity	Duration/Frequency
1	Stand-sit-stand from an armchair	3 times
2	Stand-sit-stand from a kitchen chair	3 times
3	Stand-sit-stand from a toilet seat	3 times
4	Stand-sit-stand up from a bed	3 times
5	Sit-to-lay spine on a bed	3 times
6	Lay spine-roll over-lying prone on a bed	3 times
7	Vacuum floor	120 s
8	Sweep floor	120 s
9	Wash dishes in a sink	120 s
10	Clean dining table/worktop	120 s
11	Ascend and descend stairs	3 times
12	Open a closed door, walkthrough and close the door	3 times
13	Stand still for 10 s and then get in and out of a car seat	3 times
14	Walk with a shopping bag/handbag	60 s
15	While standing, removing an item from your front pocket	3 times
16	While sitting, removing an item from your front pocket	3 times

**Table 3:** Characteristics of participants in Phase 3 of the study.

	Gender	Age (Years)	Duration of PD (Years)	FOGQ Score
**Participant 1**	M	85	13	14
**Participant 2**	M	70	13	15
**Participant 3**	M	78	5	16
**Participant 4**	F	70	14	14
**Participant 5**	M	62	16	16
**Participant 6**	M	63	19	15
**Participant 7**	M	79	16	14
**Participant 8**	M	72	8	12
**Participant 9**	M	67	17	18
**Participant 10**	M	60	13	16

**Table 4:** Inferred double-tap gesture characteristics.

	Tap_Acceleration [g]	Interval_Time [ms]	Tap_Width [ms]	Tap_Duration [ms]
**Min.**	4.47	134.17	1.91	17.07
**Max.**	8	435.86	20.96	83.15

**Table 5:** Latency and sensitivity performing a double-tap gesture in response to the occurrence of FoG.

	Latency Time [s]	Double-Taps Performed in Response to FoG Episodes		Sensitivity [%]
		True Positive	False Negative	
**Participant 1**	2.01 ± 1.03	11	3	78.57
**Participant 2**	1.08 ± 1.58	6	7	46.15
**Participant 3**	1.98 ± 1.21	21	1	95.45
**Participant 4**	1.26 ± 0.90	12	3	80
**Participant 5**	1.17 ± 0.82	4	1	80
**Participant 6**	0.79 ± 0.69	18	2	90
**Participant 7**	1.65 ± 0.55	5	2	71.43
**Participant 8**	1.11 ± 0.77	27	8	77.14
**Participant 9**	1.10 ± 0.78	14	7	66.67
**Participant 10**	2.34 ± 1.62	20	4	83.33
	1.42 ± 1.17 ^1^	138 ^2^	38 ^2^	76.87 ± 13.58 ^1^

^1^ Value expressed as mean ± SD of column. ^2^ Value expressed as total of column.

**Table 6:** Number and mean duration of FoG episodes removed from the analysis.

	Number of FoG Episodes	Mean Duration of FoG Episodes [s]
**Participant 1**	7	1.02 ± 0.56
**Participant 2**	7	0.59 ± 0.32
**Participant 3**	3	0.41 ± 0.28
**Participant 4**	0	0
**Participant 5**	1	0.31 ± 0.00
**Participant 6**	4	0.21 ± 0.15
**Participant 7**	7	0.88 ± 0.49
**Participant 8**	0	0
**Participant 9**	23	0.42 ± 0.26
**Participant 10**	15	1.41 ± 0.61
	67 ^2^	0.76 ± 0.58 ^1^

^1^ Value expressed as mean ± SD of column. ^2^ Value expressed as total of column.

**Table 7:** Sensitivity of double-tap detection function.

	Double-Taps Performed	Double-Taps Detection by Device			Sensitivity [%]	Specificity [%]
		TP	FN	FP		
**Participant 1**	20	20	0	0	100	100
**Participant 2**	7	7	0	0	100	100
**Participant 3**	26	24	2	0	92.31	100
**Participant 4**	14	13	1	0	92.86	100
**Participant 5**	4	4	0	0	100	100
**Participant 6**	31	28	3	0	90.32	100
**Participant 7**	7	7	0	0	100	100
**Participant 8**	31	30	1	0	96.77	100
**Participant 9**	20	20	0	0	100	100
**Participant 10**	24	22	2	0	91.67	100
	18.40 ± 9.48 ^1^	175 ^2^	9 ^2^	0 ^2^	96.39 ± 4.13 ^1^	100 ± 0 ^1^

^1^ Value expressed as mean ± SD of column. ^2^ Value expressed as total of column.

**Table 8:** Number of FoG episode occurring and their duration during Self-activated and Clinician-activated On-Demand cueing. * significant difference.

	Clinician-activated		Self-activated	
	Duration of FoG Episodes [s]	Number of FoG Episodes	Duration of FoG Episodes [s]	Number of FoG Episodes
**Participant 1**	4.01 ± 2.62	25	3.50 ± 0.94	18
**Participant 2**	1.70 ± 0.23 *	13	2.94 ± 0.67 *	13
**Participant 3**	5.50 ± 2.33	26	5.24 ± 1.53	24
**Participant 4**	3.34 ± 1.33	8	3.09 ± 1.31	11
**Participant 5**	2.47 ± 0.98	10	2.22 ± 1.27	5
**Participant 6**	2.24 ± 1.27	22	3.13 ± 1.91	22
**Participant 7**	2.07 ± 0.47	10	2.78 ± 0.83	12
**Participant 8**	2.96 ± 2.11	36	3.62 ± 1.68	27
**Participant 9**	2.10 ± 0.81	61	2.65 ± 1.51	37
**Participant 10**	4.85 ± 2.02 *	33	8.01 ± 3.18 *	34
**Mean ± SD**	3.35 ± 2.18 *	24.40 ± 16.23	4.20 ± 2.49 *	20.30 ± 10.41
